# PB.18: Factors affecting breast density assessment

**DOI:** 10.1186/bcr3518

**Published:** 2013-11-08

**Authors:** L Beattie, M Bydder, JC Sergeant, A Maxwell, N Barr, U Beetles, CRM Boggis, S Bundred, S Gadde, E Hurley, A Jain, E Lord, V Reece, M Wilson, P Stavrinos, DG Evans, A Howell, SM Astley

**Affiliations:** 1 University of Manchester Medical School, Manchester, UK; 2 Nightingale Centre and Genesis Prevention Centre, University Hospital of South Manchester, UK; 3 Centre for Imaging Sciences, University of Manchester, UK; 4 Department of Genetics, St Mary's Hospital, Manchester, UK; 5 Institute of Cancer Sciences, University of Manchester, UK

## Introduction

High breast density, where there is a relatively large proportion of fibroglandular tissue in the breast, is associated with increased risk of developing cancer. There are several methods of assessing breast density from mammograms, and as these sometimes disagree about whether density is high (or low), we have investigated potential causes of disagreement.

## Methods

A set of 6,422 mammograms with density assessed visually by two readers using Visual Analogue Scales, and volumetric breast density measured using Quantra™ and Volpara™ was obtained from the PROCAS (Predicting Risk Of Cancer At Screening) database. Cases were ranked from the highest to lowest density by each method. For each pair of methods the 20 cases with the largest discrepancy in rank, and the 20 with the smallest, were selected. Image features were recorded and compared.

## Results

The two volumetric methods were more likely to disagree when calcification was present and the inframammary fold was poorly positioned. When comparing Quantra™ to visual assessment, there were more skin folds and a higher compressed breast thickness in the discrepant cases. Comparing Volpara™ with visual assessment, there were more suboptimal inframammary folds and higher compression forces in the discrepant group.

## Conclusion

Although visual and volumetric methods are unlikely to produce similar density estimates, those ranked highly by one method should correspond to the high-density cases identified by another. Our study indicates the need for further investigation, as lack of ground truth means that in cases of disagreement it is not possible to tell which method produced better density estimates.

